# GENRE (GPU Elastic-Net REgression): A CUDA-Accelerated Package for Massively Parallel Linear Regression with Elastic-Net Regularization

**DOI:** 10.21105/joss.02644

**Published:** 2020-10-10

**Authors:** Christopher Khan, Brett Byram

**Affiliations:** 1 Vanderbilt University

## Summary

GENRE (GPU Elastic-Net REgression) is a package that allows for many instances of linear regression with elastic-net regularization to be processed in parallel on a GPU by using the C programming language and NVIDIA’s (NVIDIA Corporation, Santa Clara, CA, USA) Compute Unified Device Architecture (CUDA) parallel programming framework. Linear regression with elastic-net regularization (Zou & Hastie, 2005) is a widely utilized tool when performing model-based analyses. The basis of this method is that it allows for a combination of L1-regularization and L2-regularization to be applied to a given regression problem. Therefore, feature selection and coefficient shrinkage are performed while still allowing for the presence of groups of correlated features. The process of performing these model fits can be computationally expensive, and one of the fastest packages that is currently available is glmnet (Friedman, Hastie, & Tibshirani, 2010; Hastie & Qian, 2014; Qian, Hastie, Tibshirani, & Simon, 2013). This package provides highly efficient Fortran implementations of several different types of regression. In the case of its implementation of linear regression with elastic-net regularization, the objective function shown in (eq. 1) is minimized.


(1) $$\hat{\beta }=\underset{\beta }{\text{argmin}}\frac{1}{2N}\sum_{i=1}^{N}{\left(y_{i}-\sum_{j=1}^{P}X_{ij}\beta_{j}\right)}^{2}+\lambda \left(\alpha {\left\|\beta \right\|}_{1}\frac{\left(1-\alpha \right){\left\|\beta \right\|}_{2}^{2}}{2}\right)$$

To minimize this objective function, cyclic coordinate descent is utilized as the optimization algorithm. This algorithm consists of minimizing the objective function with respect to one model coefficient at a time. Cycling through all of the coefficients results in one iteration, and this process continues until specified convergence criteria are satisfied. As previously stated, glmnet is highly efficient for single model fits, but performing thousands of these fits will still require significant computational time due to each one being executed in a serial fashion on a CPU. However, by using GENRE to perform massively parallel processing on a GPU, a significant speedup can potentially be achieved. This is due to the fact that modern GPUs consist of thousands of computational cores that can be utilized. Moreover, although the processing in GENRE is performed using the C programming language and CUDA, a MEX-interface is included to allow for this code to be called within the MATLAB (The MathWorks, Inc., Natick, MA, USA) programming language for convenience. This also means that with modification, the MEX-interface can be replaced with another interface if it is desired to call the C/CUDA code in another language, or the C/CUDA code can be utilized without an interface. Note that other packages have been developed that can utilize GPUs for linear regression with elastic-net regularization, such as H2O4GPU (“H2O4GPU,” 2020). However, for this application, these packages typically focus on performing parallel computations on the GPU for one model fit at a time in order to achieve acceleration when compared to a serial CPU implementation. For GENRE, the computations for a single model fit are not parallelized on the GPU. Instead, many model fits on the GPU are executed in parallel, where each model fit is performed by one computational thread.

## Statement of Need

The core motivation for developing GENRE was that many of the available packages for performing linear regression with elastic-net regularization focus on achieving high performance in terms of computational time or resource consumption for single model fits. However, they often do not address the case in which there is a need to perform many model fits in parallel. For example, the research project that laid the foundation for GENRE involved performing ultrasound image reconstruction using an algorithm called Aperture Domain Model Image RE-construction (ADMIRE) (Byram, Dei, Tierney, & Dumont, 2015; Byram & Jakovljevic, 2014; Dei & Byram, 2017). This algorithm is computationally expensive due to the fact that in one stage, it requires thousands of instances of linear regression with elastic-net regularization to be performed in order to fit models of ultrasound data. When this algorithm was implemented on a CPU, it typically required an amount of time that was on the scale of minutes to reconstruct one ultrasound image. The primary bottleneck was performing all of the required model fits due to the fact that a custom C implementation of cyclic coordinate descent was used to compute each fit serially. However, a GPU implementation of the algorithm was developed, and this implementation provided a speedup of over two orders of magnitude, which allowed for multiple ultrasound images to be reconstructed per second. For example, on a computer containing dual Intel (Intel Corporation, Santa Clara, CA) Xeon Silver 4114 CPUs @ 2.20 GHz with 10 cores each along with an NVIDIA GeForce GTX 1080 Ti GPU and an NVIDIA GeForce RTX 2080 Ti GPU, the CPU implementation of ADMIRE had an average processing time of 94.326 ± 0.437 seconds for one frame of ultrasound channel data while the GPU implementation had an average processing time of 0.436 ± 0.001 seconds. The average processing time was obtained for each case by taking the average of 10 runs for the same dataset, and timing was performed using MATLAB’s built-in timing capabilities. The 2080 Ti GPU was used to perform GPU processing, and the number of processing threads was set to 1 for the CPU implementation. The main contributor to this speedup was the fact that the model fits were performed in parallel on the GPU. For this particular case, 152,832 model fits were performed. Note that double precision was used for the CPU implementation while single precision was utilized for the GPU implementation due to the fact there is typically a performance penalty when using double precision on a GPU. Moreover, for the CPU implementation, MATLAB was used, and a MEX-file was used to call the C implementation of cyclic coordinate descent for the model fitting stage. In addition, note that one additional optimization when performing the model fits on the GPU in the case of ADMIRE is that groups of model fits can use the same model matrix, which allows for improved coalesced memory access and GPU memory bandwidth use. This particular optimization is not used by GENRE.

Aside from this application, there are a number of other applications that can potentially benefit from having the ability to perform model fits in a massively parallel fashion, which is why the code was developed into a package. For example, linear regression with elastic-net regularization has been applied to the field of genomics in order to develop predictive models that utilize genetic markers (Ogutu, Schulz-Streeck, & Piepho, 2012; Waldmann, Mészáros, Gredler, Fuerst, & Sölkner, 2013). In addition, like ADMIRE, there are a variety of other signal processing applications. For example, this regression method has been used to create models of functional magnetic resonance imaging data in order to predict the mental states of subjects and provide insight into neural activity (Carroll, Cecchi, Rish, Garg, & Rao, 2009). Moreover, another signal processing example is that linear regression models with elastic-net regularization have been used in combination with hidden Markov random field segmentation to perform computed tomography estimation for the purposes of magnetic resonance imaging-based attenuation correction for positron emission tomography/magnetic resonance imaging (Chen et al., 2014). Now, through the use of GENRE, it is possible to reduce the amount of processing time that is required in each of the aforementioned examples by computing the models in parallel for each case.
